# Household dietary diversity and associated factors among residents of finote selam town, north west Ethiopia: a cross sectional study

**DOI:** 10.1186/s40795-017-0148-0

**Published:** 2017-03-21

**Authors:** Getnet Mekuria, Yalewsew Wubneh, Tilahun Tewabe

**Affiliations:** 1 Department of Applied Human Nutrition, Bahir Dar Institute of Technology, Bahir Dar University, Bahir Dar, Ethiopia; 2 World Vision Ethiopia, Finote Selam, Ethiopia; 3 Department of Nursing, College of Medicine and Health Sciences, Bahir Dar University, Bahir Dar, Ethiopia

**Keywords:** Household dietary diversity, Finote Selam Town

## Abstract

**Background:**

Monotonous low quality diets are the norm in resource-limited environments across the globe. Dietary diversity is a challenge for rural communities in developing countries. Their diets are based on starchy staples with inadequate animal products, fresh fruits and vegetables.

**Methods:**

A community based cross sectional study was conducted from August 22–30, 2015 at Finote Selam Town. Systematic random sampling technique was used to select 400 households. Data were collected using pretested and semi-structured questionnaire. Both bivariate and multivariate logistic regression analyses were carried out to identify factors associated with household dietary diversity.

**Results:**

The prevalence of low, medium and high dietary diversity scores were 11.8, 67.2 and 21% respectively. Family head (Men headed) [AOR = 4.75 (2.22, 10.16)], frequency of eating [AOR = 6.06 (2.82, 13.06)] and shortage of water for cooking [AOR = 5.69 (1.39, 23.27)] were significantly associated with household dietary diversity.

**Conclusions:**

In this study the prevalence of adequate household diversity was 88.2%. Family head, frequency of eating and shortage of water for cooking were significant factors for household dietary diversity. Empowering women, increasing frequency of eating among family members and avoiding shortage of water for cooking were recommended to sustain and improve household dietary diversity.

## Background

Monotonous low quality diets are the norm in resource-limited environments across the globe. Risks for a range of micronutrient deficiencies are high when grain or tuber-based staple foods dominate and diets lack vegetables, fruits and animal-source foods. Dietary diversity is widely recognized as a key dimension of diet quality. Evidences from developed countries showed that dietary diversity is strongly associated with nutrient adequacy [1].

Dietary diversity which is defined as an increase in the variety of foods within and across food groups capable of ensuring adequate intake of essential nutrients that can promote good health, physical as well as mental development [2]. The more food groups included in daily diet the greater the likelihood of meeting nutrient requirements because all nutrients can’t be found within a single food item [3].

In developing countries, dietary diversity is a challenge for rural communities. Their diets are based on starchy staples with inadequate animal products, fresh fruits and vegetables. In countries where resources are limited, lack of access to adequate and diversified diet has been identified as one of the severe problems among poor populations that resulted various forms of nutritional problems [4]. Chronic energy deficiency, inadequate energy intake and micronutrient deficiencies are among the top priority nutritional problems that affect women of reproductive age [5].

In areas where household food security is poor, meeting minimum standards of dietary quality is another challenge in many developing country settings and it has often not given enough emphasis [6]. Now, developing countries are burdened with the ‘triple burden of malnutrition’ encompasses the three dimensions of under nutrition (wasting, stunting & underweight), micronutrient deficiencies and over nutrition. Food security policies should focus not only on calorie intake but also consumption of a diversified diet. Consumption of a diversified diet promotes the intake of different nutrients and thus prevents many diseases. Reduction in dietary diversity will lead to an increase in the proportion of malnourished people [7].

Ethiopia Socioeconomic Survey (ESS) - 2013/14 reported that cereals (rice, sorghum, barley, wheat) are the most consumed food items with 90% of all households reporting consumption of at least one of these items in any form in six of the last 7 days on average [8]. In Ethiopia, 60 and 40% of households had low and medium diet diversity scores [9]. A non-diversified diet can have negative consequences on individuals’ health, well-being and development, mainly by reducing physical capacities as well as resistance to infection. In addition, cognitive development, reproductive and even social capacities may also be impaired [10]. Therefore, the aim of this study was to assess household dietary diversity and associated factors among residents of Finote Selam Town.

## Methods

### Study setting and participants

A community based cross sectional study was conducted from August 22-30/2015 in Finote Selam Town which is located at a distance of 380 Km North-West of Addis Ababa. The town has approximately 39585 total population and 5248 households. The study populations were permanent residents of Finote Selam Town from August 22-30/2015.

The sample size was calculated by using a single population proportion formula with the following considerations; prevalence of consuming diversified diet (60.4%) from previous research conducted in Addis Ababa [11], 95% confidence level, 5% margin of error and a 5% non response rate. A total of 403 households were selected using a systematic random sampling technique from a list of all households of the town.

### Measurements

Interviewer administered questionnaire was used to collect data from respondents. First, the English version of the questionnaire was prepared, translated to Amharic and then back to English by language translators in order to check for consistency.

The respondents were asked to recall all foods eaten and beverages taken in the previous twenty-four hours prior to the interview. A scale of twelve food groups was used in assessing the dietary diversity of the respondents. A single point was given to each of the food groups consumed over the reference period giving a maximum sum total dietary diversity score of 12 points for each household.

For data collection eight diploma nurses and for supervision two Bachelor of Science nurses were recruited and trained for one day before data collection started.

### Operational definitions

- **Household**: One or more people who live in the same dwelling and also share at meals or living accommodation, and may consist of a single family or some other grouping of people.
- **Household dietary diversity**: Refers to the number of food groups consumed by household members over a 24-hour period.
- **Low household dietary diversity score**: When households consumed less than or equal to three food groups within 24 h before the survey [6].
- **Medium household dietary diversity score**: When households consumed four to six food groups within 24 h before the survey [6].
- **High household dietary diversity score**: When households consumed seven or more food groups within 24 h before the survey [6]
- **Adequate household dietary diversity**: When households consumed at least four or above food groups within 24 h before the survey.
- **Inadequate household dietary diversity**: When households consumed less than four food groups within 24 h before the survey.

### Data processing and analysis

Data were checked for completeness and entered (double entry) into Epi data version 3.1. Data were cleaned and coded using Epi data and transferred to SPSS version 20 for analysis. Descriptive statistics were used to characterize respondents using different variables of interest. First, bivariate logistic regression analysis was undertaken for each explanatory variable with the outcome variable (household dietary diversity). Variables with a p-value ≤ 0.2 on bivariate logistic regression analysis were included in the multivariate logistic regression analysis. Statistical significance was determined using odds ratio with a 95% confidence interval. Statistical significance was declared if p-value was < 0.05.

### Ethical consideration

Ethical clearance was obtained from Debre Markos University College of medicine and Health Sciences ethical review committee and permission also obtained from Finote Selam district health office as well as each kebele administration units. Informed verbal consent was obtained from the study participants and the objective of the study was explained to them. Those who were not volunter to participate are not enforced to respond. Confidentiality and privacy of collected information ensured at all levels.

## Results

### Socio-demographic characteristics of households

Of the 403 eligible respondents, 400 agreed to participate in this study, which made a response rate of 99.3%. The mean age of the respondents was 39.87 years (standard deviation, SD ±10.95). Among respondents 293 (73.2%), 72(18%) and 35(8.8%) were 19–45, 46–60 and above 60 years age group respectively (Table 1).

**Table 1:** Socio-demographic characteristics of respondents: West Gojjam Zone North west Ethiopia Finote Selam town November, 2015. (*n* = 400)

Variables	Frequency	Percent
Age (in year)		
18-45	293	73.2
46-60	72	18.0
> 60	35	8.8
Religion		
Orthodox	372	93.0
Muslim	20	5.0
Protestant	8	2.0
Residence		
Urban	284	71
Rural	116	29
Family head		
Women headed	163	40.8
Men headed	237	59.2
Marital status of respondent		
Single	35	8.8
Married	235	58.8
Divorced	61	15.2
Widowed	69	17.2
Family size		
1-2	78	19.5
3-4	190	47.5
Greater than 4	132	33
Educational status of head of the family		
Unable to read and write	126	31.5
Read and write only	77	19.3
1-8 grade	69	17.2
9-12 grade	62	15.5
Diploma and above	66	16.5
Occupation of head of the family		
Government employed	63	15.8
Private employed	23	5.8
Daily laborer	66	16.5
Farmer	99	24.7
Merchant	149	37.2

Diploma = someone who successfully completed a three year course of study from College

### Socio-economic and dietary characteristics

Two hundred fifty three (63.2%) of households were food secured. Cereals (88.6%), vegetables 43(10.7%) and animal products 3(0.7%) were commonly consumed foods (Table 2 and 3).

**Table 2:** Socio-economic characteristic of Finote Selam town residents; 2015

Variables	Frequency	Percent
Grow vegetables		
Yes	107	26.7
No	293	73.3
Farming land		
Yes	129	32.2
No	271	67.8
Irrigation scheme users		
Yes	33	8.2
No	367	91.8
Monthly income^a^		
≤ 500 birr	88	22
501-1000 birr	106	26.5
> 1000 birr	206	51.5

^a^1 Ethiopian birr = 0.05 USD

**Table 3:** Dietary characteristics of Finote Selam town residents; 2015

Variables	Frequency	Percent
Dietary diversity		
Non-diversified	47	11.8
Diversified	353	88.2
Low	47	11.8
Medium	269	67.2
High	84	21
Breakfast eaten		
Yes	382	95.5
No	18	4.5
Water shortage for cooking		
Yes	10	2.5
No	390	97.5
Food insecurity access score		
Food secure	253	63.2
Mildly food insecure	76	19
Moderately food insecure	56	14
Severe food insecure	15	3.8
Who got health education		
Yes	7	1.8
No	393	98.2
Commonly eaten foods		
Cereals	354	88.5
Vegetables	43	10.8
Animal products	3	0.8
Frequency of eating		
Two times	24	6
Three times	311	77.8
Four times and above	65	16.2

Almost all (99%) of respondents consumed cereal food groups and half (50.5%) of them also consumed vegetables (Table 4).

**Table 4:** Household dietary score of Finote Selam town; 2015

	Consumption of	Frequency(yes)	Percentage
1	ANY LOCAL FOODS, bread, rice noodles, biscuits, or any other foods made from millet, sorghum, maize, rice, wheat, or any other locally available grain?	398	99
2	Any potatoes, yams, manioc, cassava or any other foods made from roots or tubers?	48	12
3	Any vegetables?	203	50.75
4	Any fruits?	30	7.5
5	Any beef, pork, lamb, goat, rabbit wild game, chicken, duck, or other birds, liver, kidney, heart, or other organ meats?	79	19.7
6	Any eggs?	32	8
7	Any fresh or dried fish or shellfish?	1	0.2
8	Any foods made from beans, peas, lentils, or nuts?	312	77.6
9	Any cheese, yogurt, milk or other milk products?	42	10.4
10	Any foods made with oil, fat, or butter?	300	74.6
11	Any sugar or honey?	156	38.8
12	Any other foods, such as condiments, coffee, tea?	360	89.6

### Factors associated with household dietary diversity

Hosmer-Lemeshow goodness-of-fit test (*p* = 0.144) was used to assess the fitness of the model. During the bivariate logistic regression analysis; head of the family, frequency of eating per 24 h, water shortage for cooking, ownership of farming land and household food insecurity access score were significantly associated with household dietary diversity.

During the multivariate logistic regression analysis; head of the family, frequency of eating per 24 h and water shortage for cooking were significantly associated with household dietary diversity. But, ownership of farming land and household food insecurity access score were not associated with household dietary diversity. The odds of household dietary diversity is higher among men headed families [AOR = 4.75(2.22, 10.16)], frequency of eating three times and above per 24 h [AOR = 6.06(2.82, 13.06)] and no shortage of water for cooking [AOR = 5.69(1.39, 23.27)] (Table 5).

**Table 5:** Factors associated with household dietary diversity of Finote Selam Town residents; 2015

Variables	Dietary diversity		COR 95% CI	AOR 95% CI
	Diversified	Non-diversified		
Head of the family				
Women headed	127(31.8)	36(9.0)	1.00	1.00
Men headed	226(56.5)	11(2.8)	5.82(2.87,11.84)	4.75(2.22,10.16)
Frequency of eating				
2 times	27(6.8)	18(4.5)	1.00	1.00
3 times and above	326(81.5)	29(7.2)	7.49(3.69,15.19)	6.06(2.82,13.06)
Water shortage				
Yes	5(1.2)	5(1.2)	1.00	1.00
No	348(87.0)	42(10.5)	8.29(2.30,29.81)	5.69(1.39,23.27)
Ownership of farming land				
Yes	123(30.8)	6(1.5)	3.65(1.51, 8.85)	
No	230(57.5)	41(10.2)	1.00	
HHFIAS				
Food secured	236(59.0)	17(4.2)	3.56(1.89, 6.72)	
Food insecure	117(29.2)	30(7.5)	1.00	

## Discussion

This community based cross- sectional study assessed household dietary diversity and associated factors in Amhara region, West Gojjam zone, Finote Selam town in 2015. The major goal of dietary diversity is to promote households to consume diversified diets rather than consuming monotonous diets throughout 24 h.

The results of this study showed that low, medium and high household dietary diversity scores in Finote Selam town were found to be 11.8, 67.2 and 21% respectively. Previous study (Zinet NH, 2013) conducted in Addis Ababa Ethiopia reported that 5.9 60.4 and 33.7% of the households had low, medium and high Dietary Diversity Scores (DDS) respectively [11]. Belachew T and Yemane T [12] reported that 39.7% of people 40 years and above in Jimma town, Southwest Ethiopia had non-diversified diet. Misker D., Misker B and Ayele G [13] revealed that 65.7 and 34.3% of the households had low and high dietary diversity scores. Taruvinga A, Muchenje V and Mushunje A [14] revealed that 29.3, 35.9 and 34.8% of rural households had low, medium and high dietary diversity scores. Rasaki SA [15] reported that 16.5 and 83% of the respondents had low and average/medium DDS. Sarkar S [16] reported that 39, 40 and 21% of the households have low, medium and high dietary diversity scores respectively. These differences might be due to variations in socio-economic status, agro-ecology, culture, time etc. In addition, the higher prevalence of adequate household dietary diversity in the study area is due to the presence of religious festival among orthodox Christian community during data collection period.

Head of the family is among various socio-demographic factors that shows significant association with household dietary diversity in this study. The odds of having adequate household dietary diversity is 4.75 times higher among men headed families compared to women headed. This has similarities with study findings from Amatole and Nyandeni districts, South Africa [14], Mirab Abaya wereda, Southern Ethiopia [13], rural households in West Bengal, India [16], Sri Lanka [17] and Ethiopia [18]. This could be due to male headed households have more money at their disposal hence the higher dietary diversity.

Frequency of eating per 24 h is another dietary factor that has a significant association with household dietary diversity. The odds of having adequate household dietary diversity is 6.06 times higher among households who consumed food three times & above compared to households who consumed food two times. This could be explained by the fact that increasing frequency of consuming food items is one of the strategies to increase dietary diversity of households.

Availability of water for cooking is one of the prerequisites for ensuring adequate household dietary diversity. No shortage of water for cooking is significantly associated with household dietary diversity in this study. The odds of having adequate household dietary diversity is 5.69 times higher among households who didn’t have water shortage for cooking compared to households who had water shortage. This could be due to the fact that clean water is important for washing, preparing and cooking our food items.

The limitations of this study are relying on 24 h dietary recall which doesn’t show the usual dietary practice of household members and affected by religious festivals.

## Conclusions

In this study the magnitude of adequate household diversity was 88.2%. Family head, frequency of eating and shortage of water for cooking were significant factors for household dietary diversity. Empowerment of women, increasing frequency of eating among family members and avoiding shortage of water for cooking were recommended to sustain and improve household dietary diversity.

## Abbreviations

AOR: Adjusted odds ratio
CI: Confidence interval
COR: Crude odds ratio
DDS: Dietary diversity scores
FAO: Food and Agriculture Organization of the United Nations
HHFIAS: Household food insecurity access score
KM: Kilo meter
SD: Standard deviation
SPSS: Statistical package for social sciences
USD: United States of America Dollar
